# Human Mesenchymal Stromal Cells from Different Sources Diverge in Their Expression of Cell Surface Proteins and Display Distinct Differentiation Patterns

**DOI:** 10.1155/2016/5646384

**Published:** 2015-12-06

**Authors:** Kourosch C. Elahi, Gerd Klein, Meltem Avci-Adali, Karl D. Sievert, Sheila MacNeil, Wilhelm K. Aicher

**Affiliations:** ^1^KFO273, Department of Urology, University of Tübingen Hospital, 72076 Tübingen, Germany; ^2^Department of Medicine II, SCeNT, Center for Medical Research, University of Tübingen, 72072 Tübingen, Germany; ^3^Department of Thoracic, Cardiac and Vascular Surgery, University Hospital of Tübingen, 72076 Tübingen, Germany; ^4^Department of Urology & Andrology, Paracelsus Medical University, Salzburg General Hospital, 5020 Salzburg, Austria; ^5^Department of Materials Science & Engineering, Kroto Research Institute, University of Sheffield, Sheffield S3 7HQ, UK

## Abstract

When germ-free cell cultures became a laboratory routine, hopes were high for using this novel technology for treatment of diseases or replacement of cells in patients suffering from injury, inflammation, or cancer or even refreshing cells in the elderly. Today, more than 50 years after the first successful bone marrow transplantation, clinical application of hematopoietic stem cells is a routine procedure, saving the lives of many every day. However, transplanting other than hematopoietic stem and progenitor cells is still limited to a few applications, and it mainly applies to mesenchymal stromal cells (MSCs) isolated from bone marrow. But research progressed and different trials explore the clinical potential of human MSCs isolated from bone marrow but also from other tissues including adipose tissue. Recently, MSCs isolated from bone marrow (bmMSCs) were shown to be a blend of distinct cells and MSCs isolated from different tissues show besides some common features also some significant differences. This includes the expression of distinct antigens on subsets of MSCs, which was utilized recently to define and separate functionally different subsets from bulk MSCs. We therefore briefly discuss differences found in subsets of human bmMSCs and in MSCs isolated from some other sources and touch upon how this could be utilized for cell-based therapies.

## 1. Introduction

The MSCs have been described for the first time as colony forming fibroblasts (CFU-F), a rare population of cells residing in the bone marrow of guinea-pigs or mice [1, 2]. Other researchers isolated MSCs from bone marrow of rabbits [3], rats [4], pigs [5], and other species. Human bmMSCs were described in the late nineties as well [6] and at the same time a breakthrough study investigated the expression of typical cell surface markers and the proliferation and differentiation properties of human MSCs in more detail [7]. In the last 20 years, a huge number of studies investigated phenotypic features and facts of MSCs. In July 2015, a web search yielded more than 357 000 hits for the term “mesenchymal stem cell” (Google Scholar; Table 1). At the same time PubMed listed about 35 000 citations, and Web of Science listed about 134 000 publications for this term.

**Figure 1:**
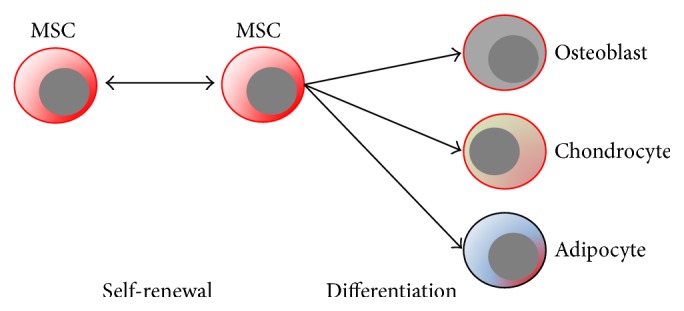
Overview on self-renewal or differentiation of stem cells in their respective stem cell niche.

When the biological properties of MSCs were explored in more detail, questions arose whether these cells met the criterion of a true* stem* cell [8]. To qualify as a stem cell, these cells must be able to self-renew, most likely by symmetric cell division to produce two daughter cells with the same stem cell qualities. At the same time, by asymmetric cell division or after specific activation, stem cells must be able to generate more mature progenitor cells or differentiated effector cells (Figure 1). Nowadays, experts agree that MSCs may generate upon appropriate stimulation quite different mature cells including osteoblasts, chondrocytes, tenocytes, adipocytes, smooth muscle cells, and stromal cells of the bone marrow [9]. They display differentiation capacities and therefore qualify as multipotent progenitor cells (Figure 1). To qualify as stem cells, self-renewal has to be shown as well [10].

Expansion of MSCs was shown to be limited to a few passages of in vitro culture and the cells underwent replicative senescence [11]. Changes in the differentiation potential of MSCs after in vitro expansion were noted and chondrogenic clones especially disappeared early on [12]. Therefore, available in vitro protocols for expansion of MSCs do not yield true* stem* cells. MSCs were also investigated for stem cell qualities in vivo. By consecutive transplantation, MSCs were able to maintain their osteogenic progenitor potential and spontaneous heterotopic ossification was observed [13]. Spontaneous generation of cartilaginous or adipose tissue was not observed experimentally after heterotopic implantation of MSCs suggesting that at least bmMSCs are self-renewing stem cells for skeletal tissue regeneration, but not stem cells for regeneration of cartilage, fat, and other tissues [13]. Therefore, in a strict sense the term mesenchymal* stem* cell applies only for osteogenesis or bone regeneration and consequently, for general purposes, the term mesenchymal* stromal* cell is preferred nowadays.

## 2. Sources for Mesenchymal Stromal Cells

**Figure 2:**
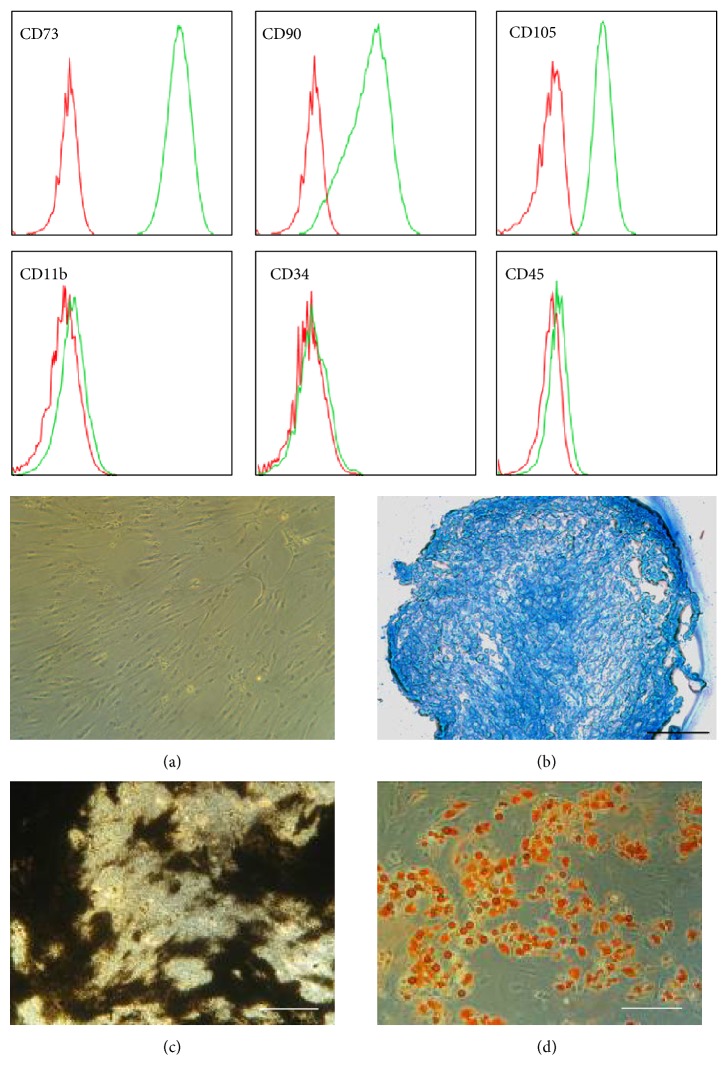
Human mesenchymal stromal cells from bone marrow (bmMSCs) are defined by their expression of CD73, CD90, and CD105, by lack of expression of a series of other cell surface markers such as CD11b, CD34, and CD45, and by a fibroblastoid appearance (a) and trilineage differentiation to generate chondrocytes (b), osteoblasts (c), or adipocytes (d) (for further details see [23]).

Mesenchymal stromal cells have been described in and have been isolated from many different adult tissues, including bone marrow, adipose tissue, inner organs, and blood vessels and from rather “young sources” such as amniotic fluid, amniotic membrane, umbilical cord, or placenta [2, 14–22]. In order to be able to discriminate MSCs from fibroblasts and other adherently growing cells, a group of experts suggested defining bmMSCs by expression of a set of cell surface markers, a trilineage differentiation potential (osteogenic, chondrogenic, and adipogenic), and fibroblast-like appearance in in vitro culture [23] (Figure 2). There is ample experimental evidence that MSCs isolated from tissues other than bone marrow share these features and generate upon stimulation osteoblasts, chondrocytes, adipocytes, and other cells in vitro as well [24–29]. However, until now, there is no experimental evidence that MSC preparations from any source contain progenitor cells that spontaneously generate fat or cartilage after transplantation to ectopic sites.

In avascular tissues such as cartilage, MSC-like cells meeting the inclusion criteria defined by the consensus conference were detected as well [31–33]. Their relation to MSCs derived from vascularized tissues, blood vessels, or serum is a matter of debate for quite some years now [34]. Still, to our momentary knowledge MSCs and MSC-like cells from different sources share characteristics including those features used to define the adult MSCs [23]. However, exploring MSCs from different sources in more detail revealed significant differences (see below).

In an adult organism and in sharp contrast to hematopoietic stem and progenitor cells [35] or spermatogonial stem cells [36], MSCs do not have stringent requirements for a stem cell niche [8], but they attach well to fibronectin, collagens, laminins, and other extracellular matrix proteins [37, 38]. MSCs were shown to appear upon adaptive transfer at several sites in a healthy recipient, and only a few cells home to bone marrow [39, 40]. Up to now, differences between the homing of MSCs to bone marrow and MSCs detected in other locations or trapped in the veins of the lung after intravenous injection of MSCs are not completely understood [40]. Moreover, the overall efficacy of homing or grafting of MSCs is low, but some MSCs applied by intravenous injection are found even at sites of injury [40]. At the same time, mobilization of MSCs occurs upon hypoxia or after injury, indicating a correlation between the migratory capacities of MSC and local wound repair [41]. A strong affinity of MSCs to a defined and specialized niche would possibly hinder the main function of these multipotent repair cells and prevent their migration to damaged sites [42, 43].

## 3. Differences in the Transcriptome of MSCs from Different Sources

Prima vista MSCs from different tissues share key characteristics such as fibroblast-like appearance in vitro, trilineage differentiation capacity, expression of certain cell surface antigens (e.g., CD73, CD90, and CD105), and lack of expression of others (e.g., CD11b, CD14, CD19, CD34, CD45, CD78, and MHC class II; Figure 2) [23, 29, 44]. Most of the studies investigating the expression of marker genes of MSCs have been performed with cells after in vitro expansion and cell culture conditions showed significant influence on the transcriptome of MSCs. For instance, bmMSCs can be isolated and explored ex vivo without time-consuming procedures and without proteolytic digestion [45]. Human bmMSCs express ex vivo the receptor for nerve growth factor (CD271), but its expression is lost by expansion of the cells in vitro [30, 46]. Comparably, expression of CD34 on MSCs from adipose tissue (atMSCs) is detected on cells ex vivo. However, it is variable and depends on the cell culture conditions [28]. Therefore, a comparison of the transcriptome of freshly isolated MSCs from different sources seems biased at least to some extent by methods employed for isolation and preparation of the cells. Furthermore, the transcriptome of MSCs from different sources is probably influenced after expansion at least to some degree by the cell culture conditions as well.

In early passages of in vitro expansion, MSCs from different sources maintain some distinct features. This statement is supported by several studies. The proliferating ability and the gene expression of human bmMSCs and atMSCs were compared [47]. The elevated proliferative capacity of atMSCs was associated with an elevated expression of transcription factor* Dickkopf* (DKK) 1. The DKK family of genes encodes soluble factors that participate in the development of mesenchymal tissues and interact with the Wnt-regulated pathway. Overall, the transcriptome of human bmMSCs and atMSCs appeared highly similar but several factors accounting for about 1% of all genes investigated are expressed at significantly different levels [47]. This highly similar gene expression profile was taken as evidence that MSCs in all tissues might be derived from a common mesenchymal precursor. Another study investigated the gene expression in neonatal MSCs isolated from umbilical cord blood (ucb-MSCs) from different donors with published gene-array data [48]. Again, bmMSCs appeared highly similar to ucb-MSCs, and the expression of five transcripts (genes) had not been reported in MSCs before (MGC3047, MGC17528, MGC3278, FLJ12442, and AGENCOURT_6683145) [48]. This study was extended by exploring the transcriptome of ucb-MSCs compared to MSCs isolated from the corresponding umbilical cord, where MSCs are found enriched in Wharton's jelly [49]. In this case, from a total of 13,699 genes investigated 1,870 were transcribed at significantly different levels representing 6% of all annotations computed [49].

**Figure 3:**
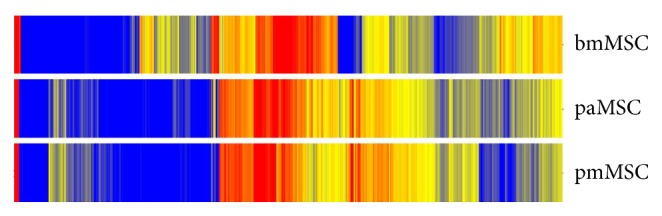
Comparing the total transcriptome of human MSCs from bone marrow (bmMSCs), from the amniotic site of human term placenta (paMSC), and from the maternal, endometrial part of human term placenta (pmMSC) in the second passage of in vitro expansion. MSCs from placenta are similar, but bmMSCs are clearly different from placenta derived MSCs.

When comparing the MSCs from amniotic sites of human term placenta with MSCs from the maternal, endometrial site of the placenta, differences were noted in the transcriptome and more than 100 genes were expressed significantly different (Figure 3). In these gene-array experiments human bmMSCs served as controls, and the differences in their transcriptome compared to both the placenta-derived MSCs from the amniotic site (paMSCs, i.e., fetal MSCs) and placenta-derived MSCs from the endometrial site (pmMSCs, i.e., maternal MSCs) are obvious even to a layperson (Figure 3). We recently confirmed that paMSCs and pmMSCs can be separated with simple methods at a sufficient efficacy as the lengths of the telomeres in paMSCs were significantly longer compared to the corresponding pmMSCs after measuring only a few samples [50]. This finding supports earlier studies reporting on higher and extended mitotic activity of neonatal MSCs, a distinct differentiation potential, and extended life span in vitro [51].

A comparison of the transcriptome of MSCs over time during in vitro expansion revealed that at least within early passages the transcriptome was stable and did not change significantly between cells in their third compared to the sixth passage of culture. At the same time, the expression of a set of core genes was preserved in bmMSCs and all neonatal MSCs were investigated, as were the differences found between bmMSCs and different MSCs from neonatal tissues [52].

Others report on significant differences in the transcriptome of bmMSCs compared to islet-derived precursor cells (IdPCs) [19] which met the minimal criteria set for bmMSCs [23]. Differences in their transcriptomes yielded distinct patterns for factors associated with gland, muscular, ectodermal, and nervous system development [19]. The differences between the transcriptomes can be associated with the differences in the origin of the cells during embryonic development: bmMSCs are derived from limb anlagen and IdPCs are derived from trunk anlagen. However, in this study bmMSCs and IdPCs were expanded in slightly different media [19]. Therefore, a bias regarding cell culture conditions cannot be excluded.

However, small differences in the transcriptome reported between MSCs from different sources do have a noticeable impact on the behaviour of the cells. Recently, we found a significant difference between bmMSCs and placenta-derived MSCs (pMSCs) not only in the expression of the cell surface molecule CD146 and the membrane-anchored alkaline phosphatase [53], but also in the expression of transcription factor Runx2 [50], a gene associated with bone development. In Runx2-targeted mice (Runx2^−/−^) endochondral ossification is completely absent as differentiation of MSCs to osteoblasts requires this transcription factor [54]. Therefore, differences in ossification observed in bmMSCs compared to pMSCs not only depend on a distinct expression of alkaline phosphatase in bmMSCs but also correlate with significant differences of regulatory factors in these cell types. At present, it can be only speculated whether the differences in expression of transcription factors influence the homing of bmMSCs versus pMSCs, atMSCs, or other MSCs [19, 50]. Of note, differences in expression of integrin components, for instance, between bmMSCs and atMSCs, have been reported, and for atMSC specific marker genes were defined [27]. Furthermore, there is compelling evidence that MSCs isolated from different sources but expanded under identical conditions share key features as defined by the so-called minimal criteria [23], but small differences in the expression of a few genes yield a significantly different type of cells when it comes to regulation of proliferation or differentiation of bmMSCs compared to MSCs from other sources [50, 52, 55]. Therefore, preselection of the best source of MSCs and possibly even preselection of subsets of MSCs may become an issue in the context of clinical applications.

Studies exploring the total transcriptome of the cells followed by bioinformatics and systems biology confirm that MSCs from different sources are very closely related cells. In this sense, the minimal criteria defined almost a decade ago seem to not only describe these cells in a correct way but also discriminate the MSCs from other progenitor cells including the hematopoietic progenitor cells or endothelial progenitor cells, found sometimes in the same niche efficiently [23].

## 4. Differences in MSC Subsets from Bone Marrow

**Figure 4:**
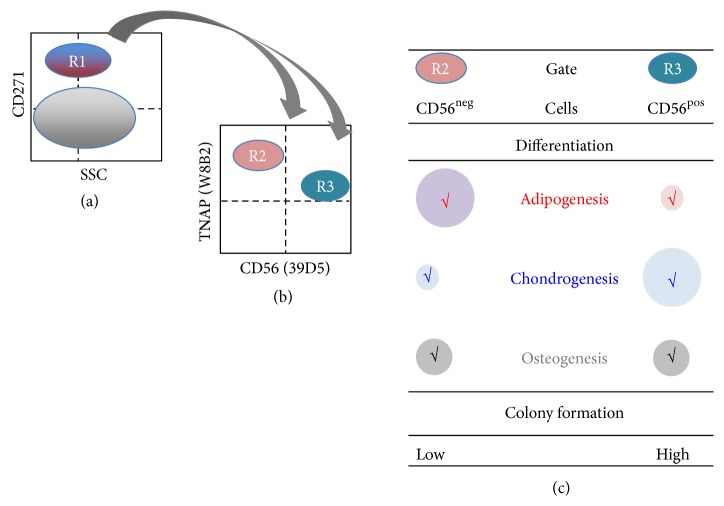
Schematic draft of the experimental strategy to define and functionally characterize subsets of human bmMSCs. MSCs were sorted by fluorescence-activated cell sorting (FACS) (left panel). From the mononuclear cells the CD271+ subset, which is gated in R1, was further subdivided by staining with two additional antibodies to TNAP and CD56 positive cells (gates R2 and R3, middle panel). The populations defined by R2 or R3 were expanded in separate cultures and their proliferation and differentiation were compared (right panel) (for further details see [30]).

In routine procedures most laboratories isolate bulk MSC populations and enrich mesenchymal stromal cells by a combination of plastic adherence followed by expansion of the cells in media preferring proliferation of MSCs [7]. Alternatively, MSCs can be isolated from bone marrow, peripheral blood, or amniotic fluid by simple gradient centrifugation and subsequently a given subset can be isolated using monoclonal antibodies to detect specific cell surface antigens expressed on some [30] but not on all MSCs [30, 45, 56]. For separation of such subsets magnetic-activated cell sorting (MACS) or fluorescence-activated cell sorting (FACS) is used. Accordingly, some of the proliferation- and differentiation-competent human bmMSCs showed expression of the receptor for nerve growth factor (CD271) and the tissue nonspecific alkaline phosphatase (TNAP), previously referred to as mesenchymal stem cell antigen- (MSCA-) 1 [30, 45]. The CD271+ subset was further subdivided using monoclonal antibodies including anti-CD56 clone 39D5. Upon expansion of the CD271+ TNAP+ CD56+ and the CD271+ TNAP+ CD56− MSC subsets in individual cultures, an interesting functional difference was observed: the CD56+ cells were proliferative and more chondrogenic, but less adipogenic compared to the corresponding CD56− cells (Figure 4). This study provided evidence that even a preselected subset of bmMSCs such as the CD271+ mesenchymal cells can be further subdivided in smaller fractions. It also supports the hypothesis that bmMSCs are ex vivo blend of cells. Accordingly, CD90+, VCAM-1+, and CD271+ bmMSCs represent another distinct bmMSC subset of cells [57, 58]. Other monoclonal antibodies defined other MSC subsets [45] and some of these antigens were characterized on MSCs in more detail recently [59]. Moreover, a population of bmMSCs with low expression of the PDGF receptor alpha (PDGFR*α*) was considered as the primary mesenchymal stromal cell [60]. Interestingly, PDGFR*α*+, CD51+, and nestin+ MSCs were shown to play a key role for generating a stem cell niche for hematopoiesis [61, 62], a feature of human bmMSCs previously associated with expression of CD146 [63].

However, nowadays not all MSC subsets as defined by monoclonal antibodies ex vivo or in vitro can be associated with functional differences, and the differences between such subsets were defined by in vitro tests. Moreover, homing experiments with bulk bmMSCs followed by detection of MSCs at sites different from bone marrow in the recipient are not definitive proof that bmMSC subsets represent cells with distinct physiological tasks as homing of MSCs can be a passive trapping in irrelevant tissues.

Due to technical limitations it is not well studied if comparable subsets of cells can be defined ex vivo in MSCs from more niches such as adipose tissue, placenta, and perivascular sites in inner organs or others. It might be that* the mesenchymal stem cell* is only present in adult bone marrow and other niches contain progenitor MSCs with a distinct tissue specificity. Alternatively,* the niche* for MSCs may be the bone marrow harbouring or attracting stem cell-like MSCs and distinct subsets of MSCs. Thus, after i.v. injection the stem cell-like MSCs and distinct MSC subsets will primarily migrate to bone marrow of the recipient. However, albeit with low efficacy, they also appear at other sites of a recipient. Inflammation and processes of tissue repair will then modulate the homing of MSCs and attract those MSC subsets needed for local regeneration.

## 5. MSC Subsets and Translation in Clinical Applications

The multipotent MSCs are an attractive cellular tool for regenerative medicine and tissue engineering [9] and more than 350 clinical trials involving MSCs are reported in the web-based registry ClinicalTrials.gov. Most studies reported focus on musculoskeletal tissues, circulation and ischemia, gastrointestinal conditions, and the nervous system [64]. Application of MSCs in clinical trials has been considered rather safe but some concerns remain [65, 66]. A recent study pointed out that in less than half of all clinical cellular trials reported to the FDA a tumorigenicity test of the cells employed was performed [67]. Other questions arise from the investigations performed during preclinical studies [68]. However, nowadays there is considerable experience with application of MSCs to treat graft-versus-host disease (GvHD). If MSCs would have a considerable risk to generate tumours or other adverse types of cells, one would expect that such significant problems come to the surface especially in patients after bone marrow transplantation and immune suppression [69].

One important concern for the safety of patients is of course the expansion of MSCs in vitro. Here an enormous variability of protocols exists and in particular studies utilizing cells from industrial sources rarely disclose the exact methods employed in cell production. For preclinical trials and more so for clinical safety and feasibility studies standardized methods or open publication of all procedures involved in cell production would be advantageous [64].

Applying bulk MSCs in a clinical context has technical advantages. The preparation of cells does not involve additional steps for selection or adaptation of the MSCs and many cells can be produced. As outlined above, MSCs prepared by standard procedures (attachment to plastic, preferred outgrowth by choice of medium) contain distinct subsets of cells. Applying, for instance, bmMSCs to treat muscular defects may yield local ossification, sometimes called heterotopic ossification [13, 70]. In this context, a selection of less osteogenic MSCs may help to avoid adverse effects. This can be done by changing the source of MSCs, for instance, by taking MSCs from adipose tissue or term placenta rather than bone marrow to regenerate muscle tissue or vasculature or by depleting the MSCs from the osteogenic subset. A significant correlation between expression of alkaline phosphatase—an enzyme needed for mineralization of bony tissue—Runx2—the key regulator of osteogenic differentiation of mesenchymal precursor cells—and CD146 was reported [22, 50] and using an antibody to CD146 allowed to separate more osteogenic from less osteogenic cells. A method to select more adipogenic or chondrogenic MSCs has been reported recently as well [30] (Figure 4). When used in a clinical context, determination or selection of MSC subsets by antibodies and flow cytometry will require standards for the technology applied [28, 37, 71], as in some cases small subsets of cells have to be defined [45]. Cell culture conditions influence the expression of cell surface antigens [28, 37], and even monoclonal antibodies yield variable results including false positive staining depending on the exact experimental conditions [72].

## 6. Conclusions

Bulk MSCs have been applied to ameliorate graft-versus-host disease or to treat autoimmune diseases for more than a decade now. Therefore, their clinical use is considered to be very safe. However, in this context it is important to recall that the route of application (i.v.) used for immunosuppression by MSCs may help to select the type of MSCs needed in the hematopoietic niche in bone marrow or in the blood system, because MSCs not matching the respective niches will be either trapped somewhere else or will be discarded. In contrast, the mechanisms contributing to the correct homing of MSCs to bone marrow or sites of tissue regeneration or other natural selective processes may not work well or not work at all in cases of a local administration of the cells, for instance, in the heart, in muscles, or in inner organs. Therefore, as there is no natural selection process for MSCs applied locally, a technical preselection could enrich the MSC subset needed clinically, as local application of a blend of MSCs may contain many unwanted cells. Some of the strategies to select or deplete subsets of MSCs have been discussed in this review. Nevertheless, today our knowledge on functional differences of MSC subsets is still not complete.

## Figures and Tables

**Table 1:** Overview of studies published regarding MSCs using the term “stem cell” or “stromal cell” in the last 20 years accessed by a web search in July 2015 (Google Scholar). It seems that the term “stem cell” became more popular although the “stemness” was only shown in a more strict sense for MSCs involved in osteogenesis and bone repair.

Search	Hits	Hits
Year	MSC as “stem” cell	MSC as “stromal” cell
1995–2000	12,000	12,100
2000–2005	41,000	21,700
2005–2010	156,000	38,200
2010–2015	148,000	33,600
